# Alteration within the Hippocampal Volume in Patients with LHON Disease—7 Tesla MRI Study

**DOI:** 10.3390/jcm10010014

**Published:** 2020-12-23

**Authors:** Cezary Grochowski, Kamil Jonak, Marcin Maciejewski, Andrzej Stępniewski, Mansur Rahnama-Hezavah

**Affiliations:** 1 Laboratory of Virtual Man, Chair of Anatomy, Medical University of Lublin, 20-439 Lublin, Poland; 2 Department of Clinical Neuropsychiatry, Medical University of Lublin, 20-439 Lublin, Poland; jonak.kamil@gmail.com; 3 Department of Biomedical Engineering, Lublin University of Technology, 20-618 Lublin, Poland; 4 Institute of Electronics and Information Technology, Lublin University of Technology, 20-618 Lublin, Poland; m.maciejewski@pollub.pl; 5 Ecotech-Complex, Maria Curie-Skłodowska University, 20-612 Lublin, Poland; andrzej.stepniewski@poczta.umcs.lublin.pl; 6 Chair and Department of Dental Surgery Medical University of Lublin, 20-081 Lublin, Poland; mansur.rahnama@umlub.pl

**Keywords:** 7 Tesla, LHON, hippocampus, blindness, Leber

## Abstract

Purpose: The aim of this study was to assess the volumetry of the hippocampus in the Leber’s hereditary optic neuropathy (LHON) of blind patients. Methods: A total of 25 patients with LHON were randomly included into the study from the national health database. A total of 15 patients were selected according to the inclusion criteria. The submillimeter segmentation of the hippocampus was based on three-dimensional spoiled gradient recalled acquisition in steady state (3D-SPGR) BRAVO 7T magnetic resonance imaging (MRI) protocol. Results: Statistical analysis revealed that compared to healthy controls (HC), LHON subjects had multiple significant differences only in the right hippocampus, including a significantly higher volume of hippocampal tail (*p* = 0.009), subiculum body (*p* = 0.018), CA1 body (*p* = 0.002), hippocampal fissure (*p* = 0.046), molecular layer hippocampus (HP) body (*p* = 0.014), CA3 body (*p* = 0.006), Granule Cell (GC) and Molecular Layer (ML) of the Dentate Gyrus (DG)–GC ML DG body (*p* = 0.003), CA4 body (*p* = 0.001), whole hippocampal body (*p* = 0.018), and the whole hippocampus volume (*p* = 0.023). Discussion: The ultra-high-field magnetic resonance imaging allowed hippocampus quality visualization and analysis, serving as a powerful in vivo diagnostic tool in the diagnostic process and LHON disease course assessment. The study confirmed previous reports regarding volumetry of hippocampus in blind individuals.

## 1. Introduction

Leber’s hereditary optic neuropathy (LHON) is an inherited mitochondrial disorder, which is a cause of progressive blindness in young men. It was described for the first time in 1871 by German ophthalmologist Theodor Leber. LHON is characterized by subacute bilateral loss of the central visual field, which is caused by focal degeneration of retinal ganglion cells and the optic nerve. More than 95% of cases of LHON subjects are caused by point mutations in the three mitochondrial DNA: T14484C, G3460A, and G11778A, which encode a subunit of the respiratory chain complex 1 [1]. The disease develops in only approximately 10% of ill women diagnosed with the mutation and in approximately 50% of men, which suggests the effect of other genetic factors on its full expression. This also suggests the role of hormonal factors and a protective effect of female sex hormones on the penetration of the disease [2,3]. With the occurrence of LHON, about 30 missense mutations concerning mitochondrial DNA are associated, and almost all of them concern the mutation of the gene encoding a subunit of nicotinamide adenine dinucleotide (NADH): ubiquinone oxidore-ductase, complex 1 of the mitochondrial respiratory chain.

The hippocampus is an essential part of the limbic system, consisting of several cell layers such as an external plexiform layer, a stratum oriens layer, a pyramidal cell layer, a stratum radiatum layer, and a stratum lacunosum-moleculare layer. The hippocampus is anatomically divided into different areas called Cornu Ammonis—CA1, CA2, CA3, and CA4. This structure receives several afferent pathways from the septal area, the entorhinal cortex, the prefrontal cortex, the premammillary region, the anterior cingulate gyrus, and the reticular formation, and provides efferent pathways to the septal area (precommissural fornix), the anterior thalamic nucleus, the hypothalamic mammillary bodies (postcommissural fornix), the entorhinal cortex, the cingulate cortex, the prefrontal cortex, and the contralateral hippocampus. The function of the hippocampus in memory and spatial navigation was thoroughly discussed in a large number of studies [4,5]. Those abilities are extremely essential in individuals with confirmed blindness due to the necessity to memorize extensive information to compensate for the incapability to instantly update spatial information. Cells responsible for the spatial navigation, interpretation of visual-based information, and orientation in specific locations were found mostly within hippocampus [6]. The para-hippocampal region was found to interpret the visual information to create map-like representations of the surrounding area, enabling navigation [7]. A study conducted on animals proved that topographical memory is based on connectivity between several brain areas including the visual cortex, the parietal and frontal regions, as well as the hippocampus [8,9]. The dependance of proper transition of visual information to the hippocampus on mental representation was also mentioned in studies analyzing brain lesions [10,11]. As a result, the compensating processes of hippocampus plasticity may be observed in such individuals.

The aim of this study was to assess the volumetry of the hippocampus in blind patients with LHON. To ensure the highest possible quality of the obtained results, we used 7T magnetic resonance to study the hippocampus. As the usage of ultra-high filed MRI allows us to acquire images with higher spatial resolution and signal-to-noise ratio (SNR) and voxel size can be smaller in comparison, i.e., 3T MRI, the images obtained with 7T MRI can provide much more accurate information about anatomical changes in the brain.

## 2. Methods

### 2.1. Subjects

Firstly, from the national health database, 25 patients with LHON were randomly included into the study. After the first selection, we checked the medical records of selected participants and excluded from further analysis those who did not meet the inclusion criteria: confirmed mitochondrial DNA mutation (11778G > A), over 18 years old, no pathological changes within the cerebrovascular system, no family history of severe neuropsychiatric disorders, capable of signing informed consent, and lastly, at least 10 years of regular education. The participants that met the exclusion criteria were removed from analysis. Exclusion criteria were: any neurodegenerative diseases, hypertension, or diabetes; patients who were pregnant or breastfeeding; and patients with any metallic implant, or suffering from claustrophobia. The scans were analyzed by an experienced neuroanatomy specialist (40 years of experience) and a clinical neuroradiologist (25 years of experience). Finally, only 18 participants were selected for further analysis; however, three of them were treated with idebenone. Due to these three people constituting too small a reference group and could disturb the distribution of the results of the rest of the studied population, they were also excluded from further analysis. The control group (HC) was selected according to the demographical data collected from the LHON group. All were non-smokers, right-handed, and had no history of persistent consumption of alcohol. In all the patients, blood pressure was assessed, and no anomalies were reported. The scans were obtained at the Ecotech Complex in Lublin, Poland, where the written consent was signed by all participants. This research was approved by the Local Committee on Medical Ethics of the Medical University of Lublin (KE-0254/23/2017) and was performed in compliance with national legislation and the Declaration of Helsinki.

### 2.2. MRI Acquisition

The spoiled gradient echo (3D-SPGR BRAVO) prepared for three-dimensional inversion recovery was obtained at Ecotech Complex Lublin from the 7T MRI with a 32-channel coil. The field of view was 220 × 220 × 180 mm and the matrix for the acquisition was 256 × 256 × 180. The images were rebuilt to a matrix of 512 × 512, giving a final voxel scale of 0.43 × 0.43 × 1 mm, with the following parameters: TE (echo time) 2.6 ms, TR (repetition time) 6.6 ms, TI (inversion time) 450 ms, 12 degree flip angle, ±31.25 kHz bandwidth. Factor 2 of parallel imaging (ARC) was used.

### 2.3. Image Analysis

**Figure 1:**
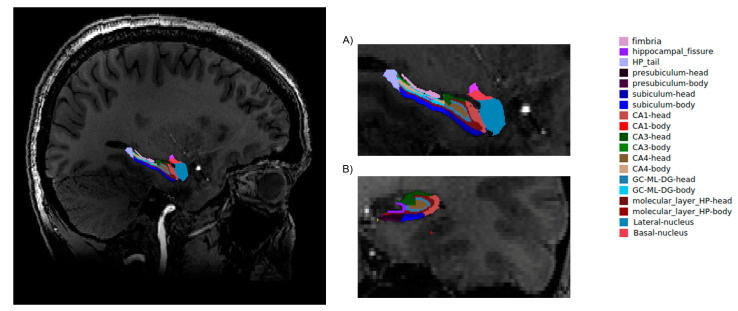
Hippocampal segmentation. T1 images of hippocampal subfields in the sagittal (**A**) and coronal (**B**) planes.

Firstly, for the correction of high-field inhomogeneity of the 7T MRI images, we applied the unified segmentation process [12] algorithms in SPM 12 (http://www.fil.ion.ucl.ac.uk/spm; MATLAB R2018A version, Mathworks, Inc., Natick, MA, USA) for each structure. A brain segmentation procedure was performed in the FreeSurfer program (http://surfer.nmr.mgh.harvard.edu/, Harvard, Boston, USA) with application of the recon-all function. During the procedure, the voxel size of the image was down-sampled to 0.5 mm3 from the native size. Recon-all as a cm flag function [13] was used to set the surface inflation number of 100. Other processing steps were similar to normal recon-all protocol (https://surfer.nmr.mgh.harvard.edu/fswiki/recon-all), such as volumetric registration, skull stripping, volumetric labelling, normalization, smoothing, segmentation, and cortical parcellation. After the initial preprocessing, the radiologist performed a quality assessment. Finally, slices contaminated by the pial surface errors, skull stripping errors, normalization problems, segmentation problems, and topological defects were removed. For the participants whose images needed re-editing, the necessary preprocessing steps were then repeated. At the final step, the hippocampus area was segmented into different subfields (hippocampal-subfields-T1 function in FreeSurfer) (Figure 1).

### 2.4. Statistical Analysis

Because of the relatively small number of participants in each of the study groups and the Shapiro–Wilk test results indicating non-compliance of our volumetric data with the normal distribution, the two-sided, non-parametric Mann–Whitney U-test for independent samples (z) was compared with the statistical significance threshold set at *p* < 0.05. In addition, the false discovery rate (FDR) was used to correct *p*-values due to multiple comparisons. During the statistical analysis, every volumetric variable was compared separately between both groups. From demographical and clinical data, differences between groups in qualitative variables, such as sex, were calculated with χ^2^. The findings were compared between volumetrically adjusted structures and selected demographic and clinical variables in the LHON subjects, such as age and period of disease, with the application of the non-parametric Spearman R test following the development of a set of volumetric metrics that significantly differentiated the classes.

## 3. Results

### 3.1. Participants

Demographical and clinical information for both groups is provided in Table 1. Neither group differed significantly in any demographical parameters. The LHON participants duration of illness lasted for about 15 years.

### 3.2. Hippocampus Volumetric Differences between Groups

The results of the volumetric analysis are shown in Table 2 for the HC and LHON groups. Statistical analysis revealed that compared to HC, LHON patients had multiple significant differences in the right hippocampus: we found a significantly higher volume of the hippocampal tail (*p* = 0.009), subiculum body (*p* = 0.018), CA1 body (*p* = 0.002), hippocampal fissure (*p* = 0.046), molecular layer HP body (*p* = 0.014), CA3 body (*p* = 0.006), GC ML DG body (*p* = 0.003), CA4 body (*p* = 0.001), whole hippocampal body (*p* = 0.018), and the whole hippocampus volume (*p* = 0.023). Additionally, LHON patients had significantly lower right fimbria volume (*p* = 0.001).

**Figure 2:**
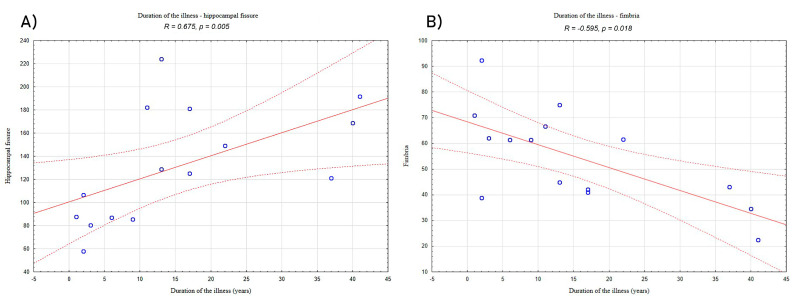
Correlation between duration of illness and (**A**) the volume of hippocampal fissure and (**B**) the volume of fimbria in the LHON patients. “°”—refers to LHON participants results; solid red line—trend line; dashed lines—confidence interval.

Analyses of relationships between volumetric data and clinical characteristics of the LHON subjects showed a significantly positive correlation between the duration of the illness and hippocampal fissure (*R* = 0.675, *p* = 0.005; Figure 2A) and a negative correlation between the duration of the illness and volume of fimbria (*R* = −0.595, *p* = 0.018; Figure 2B).

## 4. Discussion

To the best of our knowledge, this is the first study describing the volumetry of the hippocampus among LHON patients using 7 Tesla MRI. Because of the lack of vision, blind individuals are forced to create cognitive maps necessary for spatial navigation based on the other senses such as auditory sense, echolocation, proprioceptive signals, and environmental factors such as temperature and audition. Several studies mentioned the role of the hippocampus in spatial memory and navigation among sighted individuals [7,14] and Gagnon et al. proved the involvement of the hippocampus in navigation among blind subjects, including the involvement of the right hippocampus in the spatial learning processes [15].

Only a few studies discussed this matter in individuals diagnosed with blindness. A study carried out by Lepore et al. described the volume of the hippocampus among 22 blind but otherwise healthy subjects. Researchers described anatomical differences specifically within the right hippocampus, which is similar to our findings [16]. Previous studies have also shown the utility of using 7T MRI to assess changes in the hippocampus. Boutet et al. [17] presented the superiority of 7T MRI in hippocampal volume assessments in Alzheimer’s disease, whereas Springer et al. [18] reported a higher diagnostic confidence in ultra-high filed MRI in comparison to 1.5T and 3T scanners. Anterior regions (head/body) were found to have increased displacement in the blind subjects compared to sighted controls only within the right hippocampus. Fortin et al. described the alterations within the anterior portion of the hippocampus (head), linking it to the verbal memory information pathway [19]. Chebat et al. found that the volume of the right posterior hippocampus was significantly reduced compared to the sighted controls, which is contrary to our findings; however, the posterior hippocampus has been strongly linked to navigation as well as verbal memory [9,20,21]. The selectiveness of this alteration might be explained by the right hemisphere being suggested to be dominant in terms of visual and spatial skills [22]. Moreover, the involvement of the hippocampus in spatial skills and navigational tasks has been described in a great number of studies [23,24,25,26]. Lee et al. suggested that the size of the hippocampus depends on experience [27], which was later proven by Maguire et al., who described an increased volume in the posterior hippocampus in London taxi drivers as they increased in years of experience, suggesting the role of navigational experience in the structural organization of the hippocampus [20]. Patients analyzed in this study were qualified as late blind subjects (the disease occurred in adulthood), which may explain the discrepancies between studies caused by the experience-dependent plasticity of the hippocampus. This thesis was supported by several studies on late blind subjects compared to early blind subjects, suffering from different alterations patterns in cortical thickness [28,29,30] or functional connectivity density in the occipital cortex [31]. Moreover, Ma et al. found that the hippocampal tail showed more extensive resting-state functional connectivity in the blind patients [32]. 

Dahmani et al. proved the fimbria-fornix white matter volume to be associated with spatial memory as well as spatial learning, claiming that faster spatial learning is positively correlated with the right fimbria-fornix complex volume. Spatial learners showed a significant positive correlation between olfactory identification and volume of the right fimbria [33]. In this study, the negative correlation between the duration of the illness and the volume of fimbria was observed, which could suggest the involvement of the olfactory system in spatial learning among blind subjects. 

## 5. Conclusions

This is the first in vivo study applying 7T MRI to investigate changes in hippocampus volume in LHON participants. The ultra-high-field magnetic resonance imaging allowed for hippocampus quality visualization and analysis, serving as a powerful in vivo diagnostic tool in the diagnostic phase and the LHON disease course evaluation. The study confirmed previous reports of hippocampus volume in blind individuals.

## Figures and Tables

**Table 1:** Demographic and clinical data of research groups.

Variable	LHON (*n* = 15) M (SD)	HC (*n* = 15) M (SD)	*Z* Value or χ^2^	*p*
Age (years)	36.2 (14.9)	33.11 (7.17)	0.62	0.53
Education (years)	15.53 (1.81)	16 (0.95)	−1.02	0.31
Sex (% male)	86	80	0.24	0.61
Mitochondrial mutation 11778G > A (%)	100			
Duration of illness from the beginning of symptoms (months)	187.2 (159.66)			

LHON: Leber’s hereditary optic neuropathy; HC: healthy controls.

**Table 2:** Between group differences in global MST metrics (mm^3^).

Side	Structure Name	LHON (*n* = 15) M (SD)	HC (*n* = 15) M (SD)	*Z*	*p*
Right	Hippocampal tail	572.11	479.68	2.613	0.009
	Subiculum body	217.35	195.62	2.364	0.018
	CA1 body	128.75	105.62	3.069	0.002
	Subiculum head	160.91	145.29	1.618	0.106
	Hippocampal fissure	131.77	98.81	1.991	0.046
	Presubiculum head	133.82	117.52	1.908	0.056
	CA1 head	506.95	482.58	1.203	0.229
	Presubiculum body	170.24	157.91	1.037	0.300
	Parasubiculum	65.66	57.05	1.327	0.184
	Molecular layer HP head	320.88	308.35	0.871	0.384
	Molecular layer HP body	231.77	208.36	2.447	0.014
	GC ML DG head	149.42	149.05	−0.041	0.967
	CA3 body	94.81	79.03	2.738	0.006
	GC ML DG body	132.35	116.01	2.945	0.003
	CA4 head	126.23	124.82	0.083	0.934
	CA4 body	118.07	101.21	3.401	0.001
	Fimbria	54.53	78.70	−3.194	0.001
	CA3 head	122.53	120.76	0.124	0.901
	HATA	51.90	48.53	0.871	0.384
	Whole hippocampal body	1147.87	1042.45	2.364	0.018
	Whole hippocampal head	1638.30	1553.95	1.078	0.281
	Whole hippocampus	3358.28	3176.08	2.281	0.023
Left	Hippocampal tail	541.80	563.74	−0.207	0.836
	Subiculum body	216.48	207.24	1.120	0.263
	CA1 body	113.39	94.82	1.784	0.074
	Subiculum head	170.35	169.64	0.041	0.967
	Hippocampal fissure	180.47	168.89	0.498	0.619
	Presubiculum head	139.84	129.54	0.415	0.678
	CA1 head	488.79	484.37	0.166	0.868
	Presubiculum body	178.68	189.15	−0.415	0.678
	Parasubiculum	69.86	63.31	0.788	0.431
	Molecular layer HP head	315.01	311.26	0.207	0.836
	Molecular layer HP body	218.17	209.16	0.954	0.340
	GC ML DG head	145.64	145.12	0.581	0.561
	CA3 body	82.34	68.71	1.701	0.089
	GC ML DG body	125.86	120.95	0.622	0.534
	CA4 head	122.78	122.28	0.456	0.648
	CA4 body	112.13	104.03	1.452	0.147
	Fimbria	72.34	90.17	−1.949	0.051
	CA3 head	115.56	119.51	0.290	0.772
	HATA	53.48	51.52	0.249	0.803
	Whole hippocampal body	1119.39	1084.22	0.954	0.340
	Whole hippocampal head	1621.30	1596.56	0.373	0.709
	Whole hippocampus	3242.49	3144.52	0.373	0.709

CA—cornus ammonis; HATA—hippocampal amygdala transition area; HP—hippocampal; GC-ML-DG—Granule Cell (GC) and Molecular Layer (ML) of the Dentate Gyrus (DG).

## References

[1] Carelli V., Schapira A.H.V., DiMauro S. (2002). Leber’s hereditary optic neuropathy. Mitochondrial Disorders in Neurology: Blue Books of Practical Neurology.

[2] Fantini M., Asanad S., Karanjia R., Sadun A. (2019). Hormone replacement therapy in Leber’s hereditary optic neuropathy: Accelerated visual recovery in vivo. J. Curr. Ophthalmol..

[3] Hudson G., Carelli V., Horvath R., Zeviani M., Smeets H.J., Chinnery P.F. (2007). X-Inactivation patterns in females harboring mtDNA mutations that cause Leber hereditary optic neuropathy. Mol. Vis..

[4] Summerfield J.J., Lepsien J., Gitelman D.R., Mesulam M.M., Nobre A.C. (2006). Orienting Attention Based on Long-Term Memory Experience. Neuron.

[5] Rolls E.T., Xiang J.Z. (2006). Spatial view cells in the primate hippocampus andmemory recall. Rev. Neurosci..

[6] Arleo A., Gerstner W. (2000). Spatial cognition and neuro-mimetic navigation: A model of hippocampal place cell activity. Biol. Cybern..

[7] Ekstrom A.D., Kahana M.J., Caplan J.B., Fields T.A., Isham E.A., Newman E.L., Fried I. (2003). Cellular networks underlying human spatial navigation. Nature.

[8] Poucet B., Lenck-Santini P.P., Paz-Villagran V., Save E. (2003). Place cells, neocortexand spatial navigation: A short review. J. Physiol. Paris.

[9] Maguire E.A., Frackowiak R.S.J., Frith C.D. (1997). Recalling Routes around London: Activation of the Right Hippocampus in Taxi Drivers. J. Neurosci..

[10] Crane J., Milner B. (2005). What went where? Impaired object-location learning inpatients with right hippocampal lesions. Hippocampus.

[11] Feigenbaum J.D., Morris R.G. (2004). Allocentric versus egocentric spatial memoryafter unilateral temporal lobectomy in humans. Neuropsychology.

[12] Ashburner J., Friston K.J. (2001). Why Voxel-Based Morphometry Should Be Used. NeuroImage.

[13] Zaretskaya N., Fischl B., Reuter M., Renvall V., Polimeni J.R. (2018). Advantages of cortical surface reconstruction using submillimeter 7 T MEMPRAGE. NeuroImage.

[14] Bilkey D.K. (2007). Space and context in the temporal cortex. Hippocampus.

[15] Gagnon L., Schneider F.C., Siebner H.R., Paulson O.B., Kupers R., Ptito M. (2012). Activation of the hippocampal complex during tactile maze solving in congenitally blind subjects. Neuropsychol..

[16] Lepore N., Shi Y., Lepore F., Fortin M., Voss P., Chou Y.-Y., Lord C., Lassonde M., Dinov I., Toga A.W. (2009). Pattern of hippocampal shape and volume differences in blind subjects. NeuroImage.

[17] Boutet C., Chupin M., Lehéricy S., Marrakchi-Kacem L., Epelbaum S., Poupon C., Wiggins C., Vignaud A., Hasboun D., Defontaines B. (2014). Detection of volume loss in hippocampal layers in Alzheimer’s disease using 7 T MRI: A feasibility study. NeuroImage Clin..

[18] Springer E., Dymerska B., Cardoso P.L., Robinson S.D., Weisstanner C., Wiest R., Schmitt B., Trattnig S. (2016). Comparison of Routine Brain Imaging at 3 T and 7 T. Investig. Radiol..

[19] Fortin M., Voss P., Lord C., Lassonde M., Pruessner J., Saint-Amour D., Rainville C., Leporé F. (2020). Wayfinding in the blind: Hippocampal volume enhancement and spatial navigation. Brain.

[20] Maguire E.A., Gadian D.G., Johnsrude I.S., Good C.D., Ashburner J., Frackowiak R.S., Frith C.D. (2000). Navigation-related structural change in thehippocampi of taxi drivers. Proc. Natl. Acad. Sci. USA.

[21] Pruessner J.C., Li L.M., Serles W., Pruessner M., Collins D.L., Kabani N., Lupien S., Evans A.C. (2000). Volumetry of hippocampus and amygdala with high-resolution MRI andthree-dimensional analysis software: Minimizing the discrepanciesbetween laboratories. Cereb Cortex.

[22] Joseph R. (1988). The right cerebral hemisphere: Emotion, music, visual-spatial skills, body-image, dreams, and awareness. J. Clin. Psychol..

[23] Burgess N., A Maguire E., O’Keefe J. (2002). The Human Hippocampus and Spatial and Episodic Memory. Neuron.

[24] Hartley T., Maguire E.A., Spiers H.J., Burgess N. (2003). The well-worn route and the path less traveled: Distinct neural bases of route following and wayfinding in humans. Neuron.

[25] Iaria G., Petrides M., Dagher A., Pike B., Bohbot V.D. (2003). Cognitive Strategies Dependent on the Hippocampus and Caudate Nucleus in Human Navigation: Variability and Change with Practice. J. Neurosci..

[26] Mellet E., Bricogne S., Tzourio-Mazoyer N., Ghaëm O., Petit L., Zago L., Etard O., Berthoz A., Mazoyer B., Denis M. (2000). Neural Correlates of Topographic Mental Exploration: The Impact of Route versus Survey Perspective Learning. NeuroImage.

[27] Lee D.W., Miyasato L.E., Clayton N.S. (1998). Neurobiological bases of spatial learning in the natural environment: Neurogenesis and growth in the avian and mammalian hippocampus. NeuroReport.

[28] Jiang J., Zhu W., Shi F., Liu Y., Li J., Qin W., Li K., Yu C., Jiang T. (2009). Thick Visual Cortex in the Early Blind. J. Neurosci..

[29] Park H.-J., Lee J.D., Kim E.Y., Park B., Oh M.-K., Lee S., Kim J.-J. (2009). Morphological alterations in the congenital blind based on the analysis of cortical thickness and surface area. NeuroImage.

[30] Kupers R., Pietrini P., Ricciardi E., Ptito M. (2011). The Nature of Consciousness in the Visually Deprived Brain. Front. Psychol..

[31] Qin W., Xuan Y., Liu Y., Jiang T., Yu C. (2014). Functional Connectivity Density in Congenitally and Late Blind Subjects. Cereb. Cortex.

[32] Ma G., Yang D., Qin W., Liu Y., Jiang T., Yu C. (2017). Enhanced Functional Coupling of Hippocampal Sub-regions in Congenitally and Late Blind Subjects. Front. Neurosci..

[33] Dahmani L., Courcot B., Near J., Patel R., Amaral R.S.C., Chakravarty M.M., Bohbot V.D. (2020). Fimbria-Fornix Volume Is Associated with Spatial Memory and Olfactory Identification in Humans. Front. Syst. Neurosci..

